# The Impact of Drug Treatment Courts on Recovery: A Systematic Review

**DOI:** 10.1155/2013/493679

**Published:** 2013-03-21

**Authors:** Ciska Wittouck, Anne Dekkers, Brice De Ruyver, Wouter Vanderplasschen, Freya Vander Laenen

**Affiliations:** ^1^Department of Penal Law and Criminology, Institute for International Research on Criminal Policy (IRCP), Ghent University, 9000 Ghent, Belgium; ^2^Department of Orthopedagogics, Ghent University, 9000 Ghent, Belgium

## Abstract

*Introduction*. Earlier reviews regarding the effectiveness of Drug Treatment Courts (DTCs) reported a reduction in reoffending and substance use. Although substance users suffer from other difficulties than drug use and judicial issues, none of these reviews focused on outcomes or effects of DTCs on drug-related life domains, such as social relationships, employment, or health. Therefor, the present paper aims to review the impact of adult DTCs on substance use and drug-related life domains. *Method*. Primary studies were systematically searched in Web of Knowledge. Observational and controlled evaluation studies of adult DTCs were considered eligible if substance use and/or drug-related life domains were measured. *Results*. Moderately positive results were found with respect to within-program substance use. Few studies used drug-related life domains as an outcome measure and most of them yielded no effects. Employment and family relations ameliorated when specific interventions were used. *Discussion*. DTCs yield beneficial outcomes and effects regarding within-program substance use. However, evidence regarding the impact of DTCs on post-program drug and alcohol use and on other drug-related life domains is scarce. These life domains and thus QoL possibly can be improved by DTCs if specifically targeted. Future research is warranted.

## 1. Introduction

Substance use disorders (SUDs) are important public health problems in Western countries [1, 2] and are often regarded as chronic relapsing disorders [3–6]. The chronicity of SUDs is illustrated by the observation that most substance users cycle repeatedly between abstinence on the one hand and relapses into active substance use on the other hand before reaching the phase of stable recovery. Sometimes stable recovery is never reached [7]. In the past, lifelong abstinence was seen as the only indicator of recovery. In recent years, however, abstinence is seen as just one indicator of recovery. A shift towards a focus on other life domains and quality of life (QoL) as indicators for recovery from SUDs has been initiated [4, 8]. SUDs are typically associated with severe impairments in drug-related life domains such as health, wellbeing, social network, employment, and financial situation [3–6]. Difficulties in these life domains remain for several years after abstinence has been achieved, particularly in the area of employment [4]. Consequently, recovery from SUDs goes beyond abstinence, instead all associated life domains should be considered. Commitment to recovery is related to one's QoL which in its turn can be enhanced by (re)gaining and maintaining certain desired needs in life (e.g., stable housing, education and work, family, wellbeing, and stable financial situation) [9–11].

Substance users are highly prevalent in the criminal justice system [12, 13] due to substance-related offences (e.g., possession and/or consumption of drugs, driving while intoxicated and theft). Imposing judicial alternatives to imprisonment on substance using offenders contains an opportunity to divert them to community (drug) treatment services. These alternative measures are associated with improvements in drug-related life domains, next to decreases in criminal offending and substance use [14–16]. Generally, the initiation and maintenance of desistance from (drug-related) crime should be accompanied by improvement in several life domains, such as social network, employment, and housing [17–19].

At court level, Drug Treatment Courts (DTCs) were introduced to divert substance using offenders to (drug) treatment services [20]. Although DTCs differ greatly with respect to, amongst others, inclusion criteria, procedures, treatment services, and treatment providers [21], some key components can be distinguished: (1) alcohol and drug treatment and rehabilitation services are present for participants, (2) a nonadversarial approach and an ongoing judicial interaction with participants, (3) frequent alcohol and drug testing, (4) rewarding or sanctioning according to participants' compliance, (5) monitoring and evaluation of program goals during multiple court hearings, and (6) partnerships between drug court, public agencies, and community-based organizations [22].

During the past decade, several reviews have been published regarding the impact of DTCs on recidivism and substance use [21, 23–28]. In general, DTCs produce moderately beneficial results regarding recidivism, both during (within-program) [21, 23–28] and after (post-program) [23–25, 27, 28] the DTC program. These favourable outcomes apply for both drug-specific recidivism [25, 27, 28] and overall recidivism [21, 25–28]. However, Wilson and colleagues [28] concluded that DTCs have less impact on non-drug-related offences than on drug-related offences.

With respect to substance use, results are less homogenous. While the majority of reviewers concluded that substance use, as measured by drug tests, is to a certain extent reduced in DTC participants (within-program) [21, 23–25], Wilson and colleagues [28] found a negative effect of DTCs on substance use. In these review studies no information was available regarding post-program urinalysis results, since these tests are only mandatory administered within-program. Only Brown [21] does not explicitly state that the presented results are coming from within-program drug tests.

According to the GAO review [25], data on self-reported substance use within-program are contradictory, and self-reported substance use post-program did not significantly decrease. Mitchell and co-authors [27] also concluded that substance use did not significantly decline, but they did not distinguish drug test results from self-reported substance use nor within-program from post-program results.

None of the above-mentioned systematic reviews reported data on the effects of DTCs on drug-related life domains. Although the National Association of Drug Court Professionals [22, page 7] states that *“while primarily concerned with criminal activity and alcohol and other drugs use, the drug court team also needs to consider co-occurring problems such as mental illness, …, homelessness, basic educational benefits, unemployment …, spouse and family troubles.”*

Obviously, improvement in drug-related life domains plays an important role on the road to recovery and should thus be considered when evaluating DTC outcomes since these aim to reduce substance use and related criminal offending. However, previous reviews have not focused on these drug-related life domains. Therefore, the present paper aims to review the impact of DTCs on substance use and other drug-related life domains. 

## 2. Method

### 2.1. Inclusion Criteria

Studies on adult DTCs, namely, standard “DTC”, “Family Treatment Drug Court” (FTDC), “Dependency Drug Court” (DDC), “Driving Under Influence Court” (DUI), and “Driving While Intoxicated Court” (DWI) were included. FTDCs (or DDCs) and DUIs (or DWIs) are modeled on standard DTCs. The former are aimed at substance abusing parents, the latter at substance abusing drivers (especially drunk drivers). Observational or controlled evaluation studies of DTCs were considered eligible if at least one indicator of substance use and/or drug-related life domains, analogous to the subscales of the Addiction Severity Index (ASI; [29]) (namely, employment, financial situation, housing situation, physical and mental health, family and social relationships, and leisure-time activities), was reported as an outcome measure. No restrictions regarding measurement method or instruments were imposed.

### 2.2. Search Strategy and Study Selection

Web of Knowledge was consulted twice using the following search terms and Boolean operators up to December 31st, 2011: (1) a general search using *drug court* AND (*evaluation* OR effect * OR *outcome*), and (2) a specific search using *drug court* AND (*employment* OR *work* OR *income* OR *financial* OR *housing* OR *health* OR *family* OR *social* OR *leisure*). The “title only” option was marked and the search was restricted from 1989, since the first DTC was implemented in this year [20].

The general and specific Web of Knowledge searches together generated 576 hits. After removing double hits and screening title and abstract, 61 potentially relevant papers were retrieved for more detailed evaluation. These studies were screened on meeting the inclusion criteria and subsequently 45 studies were excluded after reading the full texts due to irrelevant subject, descriptive nature, a mere focus on recidivism, or secondary analysis of previously published data. Finally, 16 studies met the inclusion criteria, which are [30–45] in the reference list of this paper.

### 2.3. Data Extraction

Eligible studies were independently screened by two researchers (C. Wittouck and A. Dekkers) using a checklist to extract data on the following variables: (1) author, publication data, and country where the study was conducted, (2) DTC characteristics and procedures, (3) study design and follow-up period, (4) description of intervention and control group (if present), (5) sample size and dropout rate, (6) demographic and substance use characteristics of participants, and (7) outcome variables, measurement instruments, and study findings. Due to the heterogeneity in study methodologies and the variety in data reporting, a narrative review was regarded as most appropriate. The individual study findings were grouped according to outcome measure and were tabulated in a separate table to facilitate comparison and discussion.

## 3. Results

### 3.1. Study Characteristics

Table 1 provides an overview of included studies, according to location of DTC, study design, participant characteristics, and outcome measures.

All but one study ([32], Australia) were conducted in the USA. In half of the studies males were overrepresented (*n* = 8) [30–32, 34, 35, 38, 39, 42]. In seven studies a larger number of  female participants was present [33, 37, 40, 41, 43–45]. 

The average age of participants is 31,29 years, with a range from 24,37 [35] to 36,4 [45]. The participants are predominantly Caucasian (range 23% to 78%) [32, 43], African American (range 20% to 89%) [30, 34], or Hispanic (range 16,1% to 85,5%) [36, 39].

### 3.2. Study Quality

Study quality was assessed using the Maryland Scientific Methods Scale (MSMS) [46] and was also based on the quality assessment criteria described in the guidance for undertaking systematic reviews [47].

About half of the included studies (*n* = 7) were randomized controlled trials (RCT) [30, 34–36, 38, 42, 43]. Three studies used a quasi-experimental design (QED) [33, 41, 44], and two studies a noncontrolled pre-post design (PPD) [32, 45]. All studies, except the study of Brewster [31], specified which data collection tools were used. Often used tools were court files, judicial databases, and interviews. Sample size was mentioned in all studies, and ranged from 30 [42] to 3672 [37]. Ten studies indicated the dropout rate in their research group(s) [30, 32, 34–36, 38–40, 43, 45] with a dropout rate between 1,4% [30] over 28% [34] to 75% [32].

The follow-up period in the 16 included studies differed to a large extent. Some studies carried out a follow-up measurement after a particular time post-admission [36, 38, 41, 45]. Within these studies the follow-up period ranged from four months after baseline [45] up to three years after randomization [34]. In other studies, the outcomes were measured on several follow-up moments, both within- and post-program [30–32, 35, 39, 42, 43]. Some studies only compare post-program outcomes [33, 34, 37, 40, 44].

### 3.3. Effects on Drug Use, Alcohol Use, and Other Drug-Related Life Domains

In Table 2 the individual study findings are displayed according to study design and outcome measure. Outcome measures were drug use, alcohol use, family and social relationships, employment and income, and mental and physical health. None of the included studies reported on housing situation or leisure-time activities as outcome measures. Five studies using a RCT [35, 36, 38, 42, 43] and one study using a QED [41] were treated as observational studies since they randomized between a standard DTC and an “enhanced” DTC. Some studies only reported on post-program outcomes regarding drug use and family reunification but were still included in the review, since it could be assumed that all participants were drug users at baseline or risked losing parental rights at baseline [33, 34, 37, 40, 44].

Substance use significantly improved over time in almost all studies, regardless of study design. With respect to other drug-related life domains, the difference in results between observational studies and studies using a comparison condition is very obvious. All observational studies, except one [35], conclude that DTC participants improved on all these drug-related life domains from pre- to post-test. These beneficial results were not found by controlled studies. Most of the latter types of studies only found favourable results for DTCs on drug and alcohol use. Only FTDCs found beneficial results regarding family and social relationships.

#### 3.3.1. Effects on Drug Use

In the majority of the included studies (*n* = 10, participants *n* = 2390) illicit drug use was an outcome measure. In the Freeman study [32], illicit drug use was measured indirectly by looking into self-reported weekly spending. Weekly spending significantly decreased while weekly legal income did not change significantly, indicating a reduction in drug use over time. In the other studies drug use was measured directly either by urine test results [30, 31, 42] or self-reported data on drug use [34, 38, 45] or both [35, 39, 43].

Self-reported data on drug use showed positive results for DTC participants within-program [35, 43, 45], at discharge [39] and post-program [34, 35, 39, 43]. In addition, DTC participants reported significantly less illicit drug use than drug using offenders diverted to standard adjudication [34] and methamphetamine-dependent outpatients with no drug court supervision [39]. Leukefeld and colleagues [38] found that drug use decreased for all study groups. However, the study's high upgrading group performed significantly better than the drug court as usual and the low upgrading group.

Urine test results also showed beneficial outcomes for DTC participants during the DTC trajectory [39, 42, 43]. Only one study reported on urine test results after a DTC program. Specifically, Dakof and colleagues [43] found that mothers were less likely to provide a positive urine test after the DDC program ended. Deschenes et al. [30] found that about half of DTC participants as well as probationers delivered a positive drug test within-program. Significant differences between these two study groups emerged when type of drugs was considered. Probationers tested significantly more positive for cocaine and heroin, whereas drug court participants were significantly more likely to test positive for marijuana.

Not all studies concluded unanimously positive. Although both DTC study groups in the Marlowe et al. study [35] significantly improved with respect to self-reported drug problems, these results were not mirrored by the urine drug screen results. Positive urine drug screens increased, although not significantly, over time for both conditions. Most of these positive drug tests were related to cannabis use. But then, the vast majority of study participants were cannabis users at baseline. Brewster [31] found that, although the rate of positive drug tests was lower for DTC participants in comparison with probation participants, a comparable amount of participants from both study groups remained drug-free throughout the study period. Possibly, the higher amount of positive drug tests among probationers can be attributed to drug testing at irregular intervals (e.g. in case of suspicion) of drug use), while testing of DTC participants at regular intervals is standard practice.

#### 3.3.2. Effects on Alcohol Use

Only six studies (participants *n* = 1419) included alcohol use as an outcome measure. Eibner and colleagues [36] focused exclusively on alcohol use since they evaluated a DUI court. All six studies found that DTC participants reported significantly less alcohol use over time. DTC participants as well as outpatients with no DTC supervision, both suffering from methamphetamine dependence, reported significantly less alcohol use over time [39].

#### 3.3.3. Effects on Family and Social Relationships

In ten of the included studies (participants *n* = 6207) family and social relationships were used as an outcome measure. While in some studies social functioning [32], family functioning, and parenting practices [41, 43] of DTC participants significantly improved over time, other studies found no difference regarding family and social relationships [35].

In comparison with non-DTC methamphetamine-dependent outpatients [39] and drug using offenders processed through standard adjudication [34], family and social relations of DTC participants improved significantly.

Slightly more than half (*n* = 6, participants *n* = 5512) of these ten studies evaluated FTDC [33, 37, 40, 41, 43, 44]. Dakof and colleagues [41, 43] found that FTDC interventions can positively influence child welfare outcomes. Besides, at follow-up, family reunification was significantly more likely in FTDC cases than in cases processed through traditional child welfare services [37, 40, 44], except in the Ashford study [33]. Children of parents managed by traditional child welfare services were significantly more likely to be allocated to out-of-home-placement [37] and to reach permanency faster [40, 44]. Ashford [33], on the other hand, found that FTDC children reached a permanency decision sooner than children in traditional child welfare services. Furthermore, time to family reunification was significantly longer for children in traditional child welfare services than children in the FTDC group [40]. In addition, Burrus and colleagues [44] found that FTDC children spent less time in nonkinship foster care. Surprisingly, Boles and colleagues [37] also found that DDC participants had a higher re-entry rate to out-of-home care after family reunification than comparison participants. When outcomes of these re-entries were examined, however, it was found that all these comparison children moved to permanency, while almost one third of DDC children were subsequently reunified with their parents.

#### 3.3.4. Effects on Employment and Income

About half of the included studies (*n* = 7, participants *n* = 2048) used employment as an outcome measure, and two of these studies also reported on income. Only two studies found beneficial outcomes for DTC participants regarding employment situation. Dakof and co-authors [43] found that employment problems of mothers in DDC significantly decreased. In their evaluation of an enhanced employment intervention, Leukefeld and colleagues [38] found that high upgrading participants reported significantly more full-time employment, significantly less unemployment, and more days of working when compared to both the no-intervention and the low upgrading group. Participants in the no-intervention group worked significantly more in the past 30 days than those in the low upgrading group. In concordance with the above-mentioned results, participants in the high upgrading group reported more income from a legitimate job in the past year than those in the low upgrading group. The no-intervention group did not differ from either of the upgrading groups.

In the Marlowe et al. [35] study, reported employment problems were not significantly reduced over time for participants from both DTC conditions. Furthermore, employment situation of drug court participants did not differ significantly when compared to non-drug court participants with methamphetamine dependence [39], standard adjudication [34], and probationers [31]. Deschenes and colleagues [30] even concluded that probationers were significantly more likely to be employed than drug court participants. However, during the follow-up period, DTC participants were significantly more likely than probationers to be involved in counselling and outpatient treatment sessions [30]. Although Gottfredson and co-authors [34] did not find a significant difference regarding employment situation between drug court participants and drug offenders processed through traditional adjudication, they did find the former relying on welfare significantly less than the latter, which could be due to a reduction in money spent on drug use since drug use decreased significantly in DTC participants.

#### 3.3.5. Effects on Mental and Physical Health

In only five studies (participants *n* = 757) the effects of drug treatment court on mental and physical health was examined. Mental health [32, 43], general health and vitality [32] of DTC participants significantly increased over time, while medical problems [35, 43], bodily pain, and emotional and physical role limits [32] of DTC participants significantly decreased over time. Marlowe and colleagues [35], however, did not find improvement for mental health and Freeman [32] found no improvement regarding physical functioning.

Furthermore, no differences were found regarding mental and physical health between DTC participants and non-DTC methamphetamine-dependent outpatients [39] nor between DCT participants and drug using offenders processed through standard adjudication [34].

## 4. Discussion

The results of the present paper show that, in general, DTCs appear to produce a decrease of illicit drug use in substance using offenders during a DTC trajectory, which was also found by earlier review studies [21, 23–25]. DTC participants, who did not reach abstinence, often moved away from more harmful substances as heroin to less harmful substances as marijuana [30]. Little information is currently available on the long-term effects of DTCs on illicit drug use. Although long-term self-report data show promising results, information from urinalysis confirming these results is lacking. Alcohol use of DTC participants also decreased. Surprisingly alcohol use was an outcome measure in only half of the included studies, given the well-established link between drug and alcohol use [48–51].

Although the importance of drug-related life domains, for example employment and housing, is recognized in the recovery and desistance literature [9–11, 14, 15, 17–19], only a small amount of DTC evaluation studies focusing on these outcomes were found. The lack of empirical data on the effects of DTCs on drug-related life domains might be explained in two ways. Or DTCs focus predominantly on substance use and drug-related crime which results in a lack of attention to other drug-related life domains. Or DTC research has not yet caught up with the state of the art on recovery, resulting in a lack of focus on these drug-related life domains. Consequently, DTC research should broaden its focus and systematically record information on DTC interventions aimed at these drug-related life domains, and study the short-term and long-term effects of DTCs on these life domains and QoL of DTC participants. Subsequently, evidence-based recommendations can be made in order to improve DTC policy and practice.

As for the studies that do focus on drug-related life domains, observational studies found beneficial results, which were not *consistently* demonstrated by (randomized) controlled studies. As opposed to standard adult DTCs, FTDCs do appear to positively influence child welfare outcomes (e.g., family reunification) [37, 40, 44]. Next, Leukefeld and colleagues [38] found that DTC participants who received an enhanced employment intervention aimed at obtaining, maintaining, and upgrading employment and attended sufficient sessions, had more beneficial outcomes regarding their work situation (e.g., more full-time employment and less unemployment).

Following the (moderately) positive results of DTCs on substance use, of FTDCs on child welfare outcomes and of the DTC with an enhanced employment intervention on work situation, it can be hypothesized that providing specific interventions for each of the drug-related life domains will beneficially affect these domains. Indeed, considering the chronicity of SUDs and the complexity of associated problems [3–7], one cannot expect that a mere focus on substance use as such will automatically entail improvements in other life domains. Moreover, by offering interventions specifically aimed at improving drug-related life domains, attention is given to outcomes which are reported as desired by drug users themselves. In other words, such interventions start from drugs users' own experiences and expectations (e.g., QoL) [52]. After all, the recovery model is a widely used and accepted approach for supporting persons with mental health and/or addiction problems, ultimately aimed at improving QoL [9, 53, 54]. When substance users experience progress in those drug-related life domains they consider important, and subsequently in their QoL, their drug use and criminal offending could be positively influenced.

No information was available regarding the effects of DTCs on housing situation or leisure-time activities, although research shows that moving away from drug-using friends and acquaintances supports the maintenance of abstinence [17] and permanent housing is associated with a reduction in recidivism [14, 15]. In addition, non-substance-related leisure-time activities contribute to a higher QoL [55].

Some limitations of the present paper should be addressed. First, one should be cautious when generalizing the results of the present paper. The restriction of the search strategy to peer reviewed publications could have induced publication bias, since often only favorable results are published. Although peer review also guarantees some form of quality control. Mitchell and colleagues [27, p. 69] also found that the results of published and unpublished studies on the effects of DTCs are “roughly similar.” Next, only a small number of individual studies reporting on the effects of DTCs on drug use, alcohol use, and/or drug-related life domains were available. The included studies are also marked with substantial heterogeneity regarding methodology, sample size, and measurement instruments. Comparability of individual study results is further compromised since comparability between DTCs from different jurisdictions is low (e.g., inclusion criteria, procedures, treatment services, and treatment providers) [21]. Evaluations are thus very site-specific, which gives little insight into the overall effectiveness of the system [56]. In fact, a general DTC framework and general DTC theoretical underpinnings are lacking [56, 57]. Generalizability is further limited since beneficial outcomes are more prevalent in DTC graduates than in dropouts [25, 27], and in general about half of DTC participants drop out of the trajectory [21, 23–25]. The dropout rate varied greatly between the included studies, and intent-to-treat analyses were not carried out or were impossible to carry out. In addition, the included studies were almost exclusively USA based. The adoption of USA DTC practices and the generalizability of USA DTC results to other countries, especially European, is problematic because of fundamental differences in the law system. Second, RCTs, which receive the highest score on the Maryland Scientific Methods Scale [46], are rare in evaluating DTC's effectiveness. In the present review less than half of the 16 included studies used a RCT, and only two of these RCTs randomly assigned participants to DTC or another kind of judicial processing. The observational and quasi-experimental studies do not allow to conclude that the observed beneficial outcomes are attributable to DTCs, since improvement due to confounding factors cannot be ruled out. Finally, to our knowledge, the longest follow-up period in DTC evaluation studies is three years [34]. Empirical data on the long-term effects of DTCs on drug-related life domains and substance use (and criminal offending) is thus lacking since studies with extended follow-up periods are non-existent.

To conclude, although one should consider the abovementioned limitations when generalizing the present paper's results, some important conclusions should be highlighted. First, through a dominant focus on substance use and criminal offending, DTCs and DTC research possibly suffer from a lack of attention and interest for other drug-related life domains and QoL of substance users. Second, these life domains can be improved as long as they are addressed. Consequently, DTC policy and practice should be adapted according to the recent findings of recovery and desistance research by focusing on improvement in drug-related life domains and by targeting these domains using specific interventions thus improving QoL of substance using offenders. In addition, each DTC trajectory should be tailored to the unique problems a DTC client faces, herewith assuring a more individual approach. As research has shown that great interpersonal variability exists between DTC participants, and that the effectiveness of DTCs differs according to these differences [38, 58, 59]. Finally, it can be expected that a decrease in substance use and criminal offending results from better life circumstances for substance users. Therefor, future research on the effectiveness of DTCs should use a more comprehensive focus and study the short-term and long-term effects of DTCs on drug-related life domains and QoL next to the effects on substance use and criminal offending, which are, after all, socially desirable outcomes [52]. 

## Figures and Tables

**Table 1:** Overview of included studies, according to location of DTC, study design, study groups, participant characteristics, follow-up period and outcome measures.

Study	Location	Study design	Study group(s)	Participant characteristics	Follow-up period	Outcome measures
Deschenes et al. (1995) [30]	USA	RCT	DTC (*n* = 176) versus Probation (*n* = 454)	Mean age at current conviction 30 years Male (75%) Alcohol and drug use: after alcohol, the primary drug of choice was marijuana followed by cocaine.	Before, during and after program up till 12 months after admission	Substance use Employment

Brewster (2001) [31]	USA	Non-randomized controlled trail	DTC (*n* = 184) versus Probation (*n* = 51)	No information on age Male (DTC: 84%, probation: 78%) Drug use: 49% DTC versus 43% cocaine or crack and 61% DTC versus 39% marijuana as one of their top three drugs of choice 47% DTC versus 35% marijuana primary choice 34% DTC versus 26% cocaine primary choice	BL Discharge 30 & 90 days post-discharge and other measurements at 3, 6, 9, 12 months FU	Vocational status Substance use

Freeman (2003) [32]	AUS	Non-controlled pre post design	DTC (*n* = 51)	Mean age 27 years Male (82%) Drug of choice: 82% heroin, 75% reported using heroin daily	BL 4, 8 & 12 months IP	General health Mental health Bodily pain Physical functioning Social functioning Physical role limits Emotional role limits Vitality Drug use

Ashford (2004) [33]	USA	Blended quasi-experimental design with single-post program comparison	FTDC (*N* = 33) versus Traditional child welfare (*n* = 45)^1^	No information on age Female (FTDC: 82%, traditional child welfare: 73%) No information on alcohol or drug use	Up till 2 years IP	Engagement and completion rates of residential treatment Parental rights Percentage of children placed with their parents

Gottfredson et al. (2005) [34]	USA	RCT	DTC (*n* = 139 →*n* = 93 interviewed) versus Standard adjudication (*n* = 96 → 64 interviewed)	Mean age 34.8 years Male (74%) No information on alcohol or drug use	36 months after randomization	Drug use Physical health Mental health Family and social relationships Education Employment

Marlowe et al. (2005) [35]	USA	RCT	“As-needed” condition (*n* = 100) versus “Bi-weekly” condition (*n* = 100)^2^	Mean age 24.37 years Male (77%) Alcohol and drug use: abusing cannabis (74%), alcohol to intoxication (56%), sedatives (9%), cocaine/stimulants (8%), opiates (7%), or hallucinogens (7%).	Months IP 6 & 12 months post admission	Alcohol use Drug use Psychiatric Employment Family Medical

Eibner et al. [36]	USA	RCT	DUI Court (*n* BL = 139, *n* FU = 117) versus Court sanction as usual (*n* BL= 145, *n* FU = 119)	*DUI*: Mean age 36.2 years No information on gender Alcohol use: 3.6 alcohol problems index, 3 drinks a day, 7.4 drinking days per month, 2.9 days with 5+ drinks past month *Comparison group*: Mean age 34 years No information on gender Alcohol use: 3.8 alcohol problems index, 2.7 drinks a day, 8 drinking days per month, 2.9 days with 5+ drinks past month	BL 2 years after BL	Problematic drinking

Boles et al. (2007) [37]	USA	Non-randomized comparison group design Historical control	DTC (*n* = 1,291 parents and 2,097 children) versus Standard CPS and ADS Divisions services (*n* = 111 parents and their 173 children)	*DTC*: Mean age 32 years Female (70.4%) Drug of choice: 51.3% methamphetamine, 17.1% alcohol, 17.1% marijuana, 10.1% cocaine, 2.3% heroin *Comparison group*: Mean age 33.4 years Female (64.9%) Drug of choice: 44.1% methamphetamine, 18.6% alcohol, 20.3% marijuana, 10.2% cocaine, 6.8% heroin	24 mnd PP	Reunification with children

Leukefeld et al. (2007) [38]	USA	RCT (Pre-test/post-test randomized design)	The enhanced employment intervention (low upgrading *n* = 118, high upgrading *n* = 120)^3^ versus DTC (*n* = 239)	*High upgrading group:* Mean age 31.3 years Male (65%) Alcohol and drug use: years of alcohol use in lifetime 6.1, years used marijuana in lifetime 6.7, years used cocaine in lifetime 3.3, years used multiple substances in lifetime 4.5 *Low upgrading group:*Mean age 28.4 years Male (65%) Alcohol and drug use: years used alcohol in lifetime 6.5, years used marijuana in lifetime 6.7, years used cocaine in lifetime 3.7, years used multiple substances in lifetime 5 *Comparison group*: Mean age 31.3 years Male (64%) Alcohol and drug use: years used alcohol in lifetime 7, years used marijuana in lifetime 6.9, years used cocaine in lifetime 4, years used multiple substances in lifetime 5.4	BL 12 months after BL	Employment

Marinelli-Casey et al. (2008) [39]	USA	Non-randomized comparison group design	DTC (*n* = 57) versus Outpatient treatment under non drug court condition (*n* = 230)	*DTC*: Mean age 32 years Male (66.7%) Drug use: mean days of MA use in the past month 8.7 *Comparison group*: Mean age 33 years Male (58.7%) Drug use: mean days of MA use in the past month 12.6	BL Weekly IP 6 & 12 months PP	MA-use ASI (psychosocial outcomes) → legal, employment, medical, psychological, family, drug, and alcohol

Worcel et al. (2008) [40]	USA	Non-randomized comparison group design, matching	FTDC (*n* = 301) versus Traditional child welfare services (*n* = 919)	No information on age Predominantly female *FTDC*: Drug of choice: 38% methamphetamine, 18% cocaine, 11% marijuana, 26% alcohol *Comparison group*: Drug of choice: 42% methamphetamine, 13% cocaine, 10% marijuana, 24% alcohol	2 years post child welfare petition	Parent-child reunification

Dakof et al. (2009) [41]	USA	Quasi-experimental test of 80 consecutive enrollments	Engaging moms program^4^ (*n* = 43) versus FTDC (*n* = 37)	Mean age in their 30s Females only Drug use: drug of choice was mainly cocaine or crack	BL 15 months after entering program	Drug court graduation Family reunification

Marlowe et al. (2009) [42]	USA	RCT	DTC + adaptive interventions (*n* = 16) versus DTC (*n* = 14)	Mean age 27.60 years Male (77%) Alcohol and drug use: previous 30 days: use of marijuana (47%), alcohol (43%), opiates (13%) or cocaine/stimulants (6%) and multiple substances (37%)	BL IP 4 months after BL	Drug negative urine specimens

Dakof et al. (2010) [43]	USA	RCT	Engaging Moms program (*n* = 31) versus FTDC (*n* = 31)	No information on age Females only Drug use: primarily polydrug users	BL 3, 6, 12, 18 months after intake	Child welfare dispositions Substance use Mental health Parenting practices Family functioning

Burrus et al. (2011) [44]	USA	Quasi-experimental comparison group design	FTDC (*n* = 200) versus Traditional child welfare system (*n* = 200)	No information on age Female (FTCD: 98%, traditional child welfare: 100%) No information on alcohol or drug use	12 month after BL	Treatment (time to treatment, days spend in treatment, completion of at least one treatment episode) Child welfare Child welfare cost savings

Johnson et al. (2011) [45]	USA	Non-controlled pre post design	DTC (*n* = 261)	Mean age 36.4 years Females only Drug use: cocaine dependence most common SUD diagnosis (45%)	BL FU 4 months post BL	Crack use and days using crack

^1^In this study, there were two comparison groups (“treatment refusal group” and “traditional child welfare”) next to the intervention group (“FTDC”). However, comparison group “treatment refusal” and it's results were unclear, therefore this group was not included in this review.

^2^Participants were randomly assigned at intake either to attend judicial status hearings on a bi-weekly basis throughout their enrollment in drug court (“bi-weekly condition”), or to be monitored by their treatment case managers who petitioned the court for status hearings as needed in response to serious or repeated infractions (“as needed condition').

^3^Enhanced employment intervention aimed at obtaining, maintaining, and upgrading employment and attending upgrading sessions. The number of upgrading sessions attended by each participant was divided by the total number of possible upgrading sessions that a participant could have attended. The resulting percentages were then split in half, with those below the median in the low upgrading group and those above the median in the high upgrading group*.*

^4^Engaging Moms Program(EMP) was adapted for use in a family drug court context. EMP was designed to help mothers succeed in drug court by helping them comply with all court orders, including attending substance abuse and other intervention programs (e.g., domestic violence counselling, parenting classes, etc.), attending court sessions, remaining drug free, and demonstrating the capacity to parent their children. The only difference between the FTDC and EMP groups was the working relationship between the drug court caseworker and the mothers; all other aspects of the programs, including overall requirements, phases, and sanctions and rewards, were exactly the same.

**Table 2:** Individual study findings according to study design and outcomes measure*.

	Drug use	Alcohol use	Family (and social) relationships	Employment	Income	Mental health	Physical health
PPD^1^							
Johnson et al. (2011) [45]	**+**	#	#	#	#	#	#
Freeman (2003) [32]	**+**	#	**+**	#	#	**+**	**+**
RCT^2^ or QED^3^ treated as PPD							
Dakof et al. (2010) [43]	**+**	**+**	**+**	**+**		**+**	**+**
Dakof et al. (2009) [41]	#	#	**+**	#	#	#	#
Marlowe et al. (2009) [42]	**+**	#	#	#	#	#	#
Leukefeld et al. (2007) [38]	**+**	**+**	#	**+**	**+**	#	#
Eibner et al. (2006) [36]		**+**	#	#	#	#	#
Marlowe et al. (2005) [35]	+ (−)	**+**	**=**	**=**		**=**	**+**
QED							
Burrus et al. (2011) [44]	#	#	**+**	#	#	#	#
Worcel et al. (2008) [40]	#	#	**+**	#	#	#	#
Marinelli-casey et al. (2008) [39]	**+**	**=**	**=**	**=**		**=**	**=**
Boles et al. (2007) [37]	#	#	**+**	#	#	#	#
Ashford (2004) [33]	#	#	**=**	#	#	#	#
Brewster (2001) [31]	**=**	#	#	**=**	#	#	#
RCT							
Gottfredson et al. (2005) [34]	**+**	**+**	**=**	**=**	**+**	**=**	**=**
Deschenes et al. (1995) [30]	**+**	#	#	**−**	#	#	#

*A “+” indicates a significant difference in favor of the DTC, a “−” indicates a significant difference in favor of the control group, a “=” indicates no significant difference between the DTC and the control group, and “#” indicates that the outcome variable was not reported.

^1^non-controlled pre-post design.

^2^randomized controlled trial.

^3^quasi-experimental design.
